# A novel application of a bioactive material as a pit and fissure sealant: in vitro pilot study evaluating the sealing ability and penetration

**DOI:** 10.1007/s40368-022-00773-z

**Published:** 2022-12-28

**Authors:** D. Bishayi, A. Srinivasan, K. Y. Mahabala, S. Natarajan, A. Rao, A. P. Nayak

**Affiliations:** 1 Manipal College of Dental Sciences, Mangalore, Manipal Academy of Higher Education, Manipal, India; 2 Department of Pediatric and Preventive Dentistry, Manipal College of Dental Sciences, Mangalore, Manipal Academy of Higher Education, Manipal, India; 3 Department of Oral Pathology and Microbiology, Manipal College of Dental Sciences, Mangalore, Manipal Academy of Higher Education, Manipal, India

**Keywords:** Pit and fissure sealant, Microleakage, Primary prevention, Glass ionomer cement, Retention

## Abstract

**Purpose:**

To compare the sealing ability and penetration of a bioactive material used as pit and fissure sealant to those of glass ionomer sealant.

**Methods:**

This was an in vitro experimental study conducted on 20 permanent teeth. For Group I of ten teeth, ACTIVA BioACTIVE-Base/Liner was applied as a sealant on pits and fissures, and the remaining ten teeth of Group II were sealed using glass ionomer cement. After thermocycling, the apex of the teeth was sealed using composite resin and they were immersed in 1% methylene blue solution, buffered at pH 7 for 24 h. Longitudinal sections were obtained from each tooth for evaluating the sealing ability and penetration, using a binocular light microscope at 4 × magnification. The obtained data were subjected to analysis using the Chi-square test and independent *t* test.

**Results:**

The comparison of the sealing ability and sealant penetration, between the two groups, showed statistically no significant difference (*p* = 0.104 and *p* = 1.0, respectively).

**Conclusion:**

Bioactive material as a pit and fissure sealant, performed on par with glass ionomer sealant in terms of tested properties like sealing ability and penetration.

## Introduction

Dental caries is a highly prevalent oral condition that shows deleterious outcomes for each patient and the public regarding medical, social, and economic concerns (Cvikl et al. 2018). A significant number of dental caries affecting children and younger age groups is limited to the occlusal aspects of the erupting permanent molars (Carvalho 2014; Ahovuo-Saloranta et al. 2016). Pit and fissure caries constitute 44% of all caries involving the primary teeth and 80–90% in the permanent teeth (Beauchamp et al. 2008). This is because the pits and fissures are inaccessible for cleaning with the help of toothbrushes, thereby retaining plaque (Szoke 2008; Carvalho et al. 2016). The application of dental sealant forms a barrier physically on the tooth surface and lessens the growth of microbes by blocking their nutrition (Ahovuo-Saloranta et al. 2016). Sealants are not only efficient in preventing dental caries, but also reliable evidence shows that they have the potency to stop the advancement of white spot lesions (Wright et al. 2016).

The routinely used pit and fissure sealants include resin sealants and glass ionomer sealants. The disadvantages of using resin-based materials as sealants include polymerization shrinkage and moisture sensitivity. While polymerization shrinkage potentially results in microleakage, moisture sensitivity limits its usage in difficult-to-isolate cases (Kantovitz et al. 2008; Mehrabkhani et al. 2015; Bhat et al. 2013). Moreover, a stronger biofilm might be seen accumulating on resin-based materials (Yu et al. 2016). On the other hand, when used as a sealant, glass ionomer cement often fractures due to its limited strength to withstand masticatory loads (Feigal and Donly 2006). Compromised retention is another drawback (Bhat et al. 2013).

Bioactive materials are a fairly recent addition to dentistry. They are reported to release more fluoride compared to glass ionomers. Their unique chemistry not only boosts the natural remineralization process but also aids in forming a seal between the tooth and the material. Additionally, they respond to the changes in the salivary pH by taking up calcium, phosphate, and fluoride ions hence balancing the tooth’s chemical composition (Kaushik and Yadav 2017). One such recently introduced smart material is ACTIVA BioACTIVE-Base/Liner (Pulpdent, USA). According to its manufacturers, it is the perfect blend of the strength and aesthetics of composite resins as well as the beneficial properties of glass ionomer cements such as release, and recharge of calcium, phosphate, and fluoride ions, moisture tolerance, and chemical bonding (Amaireh et al. 2019).

The aforementioned properties of ACTIVA BioACTIVE-Base/Liner instigated us to evaluate its ability to be used as a pit and fissure sealant. The sealant’s penetration depth that affects the retention is another factor that determines the longevity of pit and fissure sealants (Grewal and Chopra 2008; Symons et al. 1996). Thus, this study was conducted to comparatively evaluate the sealing ability and penetration of a bioactive material used as pit and fissure sealant. The null hypothesis for the study was set as there will not be any difference in the sealing ability and penetration of a bioactive material used as a pit and fissure sealant when compared to those of GIC sealant.

## Materials and methods

This was an in vitro intergroup comparative study and was initiated following the Institutional Ethics Committee’s approval (Reference number: 21006). As this study is the first of its kind using ACTIVA BioACTIVE-Base/Liner for pit and fissure sealing, it was conducted as a pilot study, using ten samples per group for all the tested parameters.

### Specimen preparation

Twenty noncarious intact permanent teeth (molars and premolars) with deep retentive pit and fissures, extracted for orthodontic and therapeutic reasons were selected. Teeth that were cracked, fractured, attrited, filled, or already sealed were excluded. The selected teeth were stored in 10% neutral formalin until use and cleaned thoroughly using tap water just before use (Ansari et al. 2004). A fine pumice slurry, along with a rubber cup rotating at a low speed, was later used to clean the crowns of the teeth. After rigorous rinsing and air drying, the teeth were grouped at random into two sets of ten teeth each, using the toss of a coin.

#### Group I (bioactive material sealant)

Each tooth was slightly dried removing excess moisture with a cotton pellet. Care was taken not to desiccate the tooth. The tip provided by the manufacturers was placed on the ACTIVA BioACTIVE-Base/Liner (Pulpdent, USA) syringe, and the material was dispensed onto a mixing pad, which was then mixed and applied to the occlusal aspects of the tooth sample with the help of a plastic filling instrument. Applied cement was spread on the pits and fissures using a disposable fine brush. After leaving it to flow for 10 s, it was light-cured for 20 s.

#### Group II (GIC sealant)

The occlusal surface was cleaned for 20 s with GC conditioner (GC Corporation, Japan), and then rinsed. The surface was dried with a cotton pellet. Glass Ionomer cement (Fuji VII^®^, GC Corporation, Japan) was mixed as per the instructions provided by manufacturers and then applied to the occlusal surface of the tooth with the help of a plastic filling instrument. It was spread on the pits and fissures using a disposable fine brush. The cement was allowed to set, after which petroleum jelly was applied to the sealed surfaces (Ulusu et al. 2012).

Subsequent to the sealant application, all the teeth were put through 1500 thermal cycles of alternating temperatures, between 5 and 55 °C. Following sealing the tooth apex with composite resin (Tetric N Ceram, Ivoclar, Vivadent AG, Liechtenstein), two layers of nail paint were applied in such a way to leave 1 mm of sealant margin. For the next 24 h, the teeth were dipped in a 1% methylene blue solution, at pH 7 (Garg et al. 2019). Then they were washed under running water. The tooth samples were completely embedded in auto-polymerizing clear acrylic resin (i-Acryl, i-MED industries, Karnataka, India). They were then longitudinally sectioned in the bucco-lingual direction from the central fossa, using a saw-mounted diamond disc at a low speed under water coolant, hence yielding two sections of 500 µ per tooth.

### Sealing ability evaluation

The examination of dye penetration was done under a binocular microscope at 25 × magnification. Each section was photographed and then evaluated using the Ovrebo and Raadal (1990) criteria:

- Score 0: Dye penetration is absent.
- Score 1: Dye penetration is present in the part around the sealant.
- Score 2: Dye penetration is present in part below the sealant.
- Score 3: Dye penetration is present at the base of the fissure.

### Evaluation of sealant penetration

The sealant penetration, fissure morphology, and an unfilled space of each tooth were assessed at a minimum magnification of 4 × using a binocular light microscope.

**Fig. 1:**
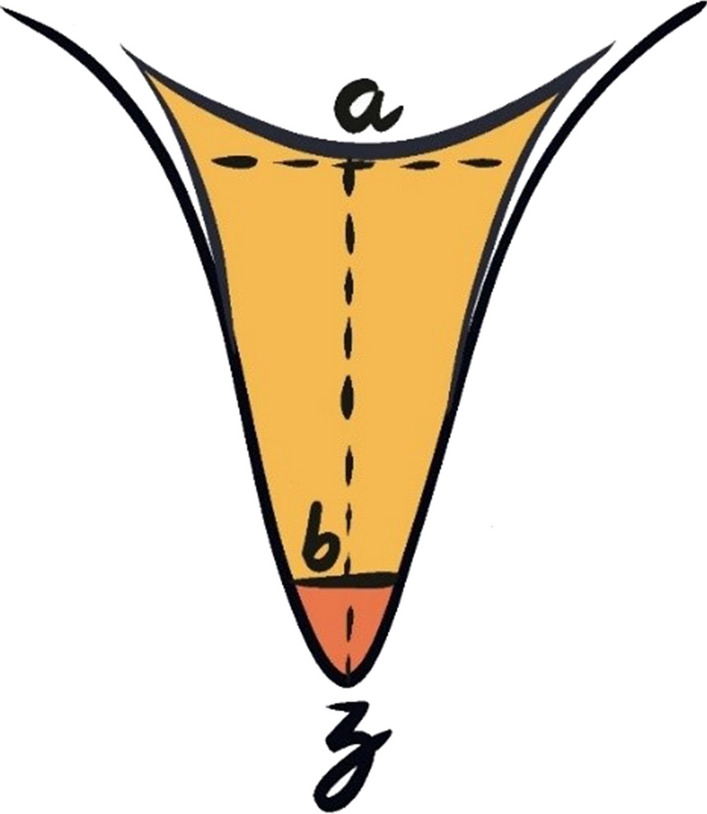
Schematic representation of the fissure with various points marked for measurement of the penetrability of the sealant with various markings as: (a) upper margin of the sealant, (b) sealant base, and (z) fissure base

The fissure morphologies found were categorized as U, V, I, and IK. The sealant penetrability and unfilled space were evaluated using the following measures (Garg et al. 2018) as derived from the points shown in Fig. 1:

1. Sealant penetration depth: The depth of the sealant penetration is measured from the deepest point of the upper margin of the sealant (a) to the sealant base (b).
2. Length of Unfilled space: The length measured (µ) between the sealant base (b) and the fissure base (z).
3. Total length of the fissure: The length measured (µ) between the deepest point on the upper margin of the sealant (a) and the fissure base (z).
4. Penetrability (%) = (sealant penetration depth/total length of fissure) × 100.

**Fig. 2:**
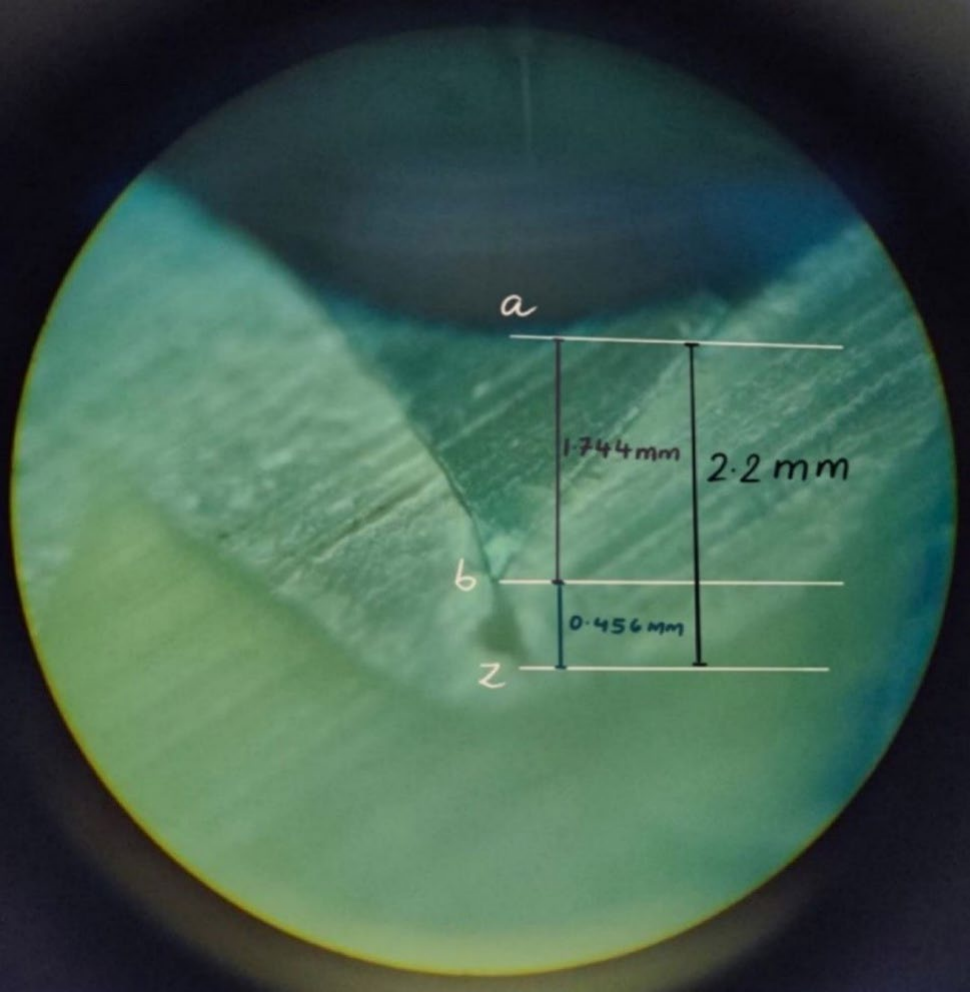
Microscopic image of a sectioned tooth with ACTIVA BioACTIVE Base/Liner applied as sealant illustrating the measurements made to check the penetrability. The fissure morphology noted here was IK type

**Fig. 3:**
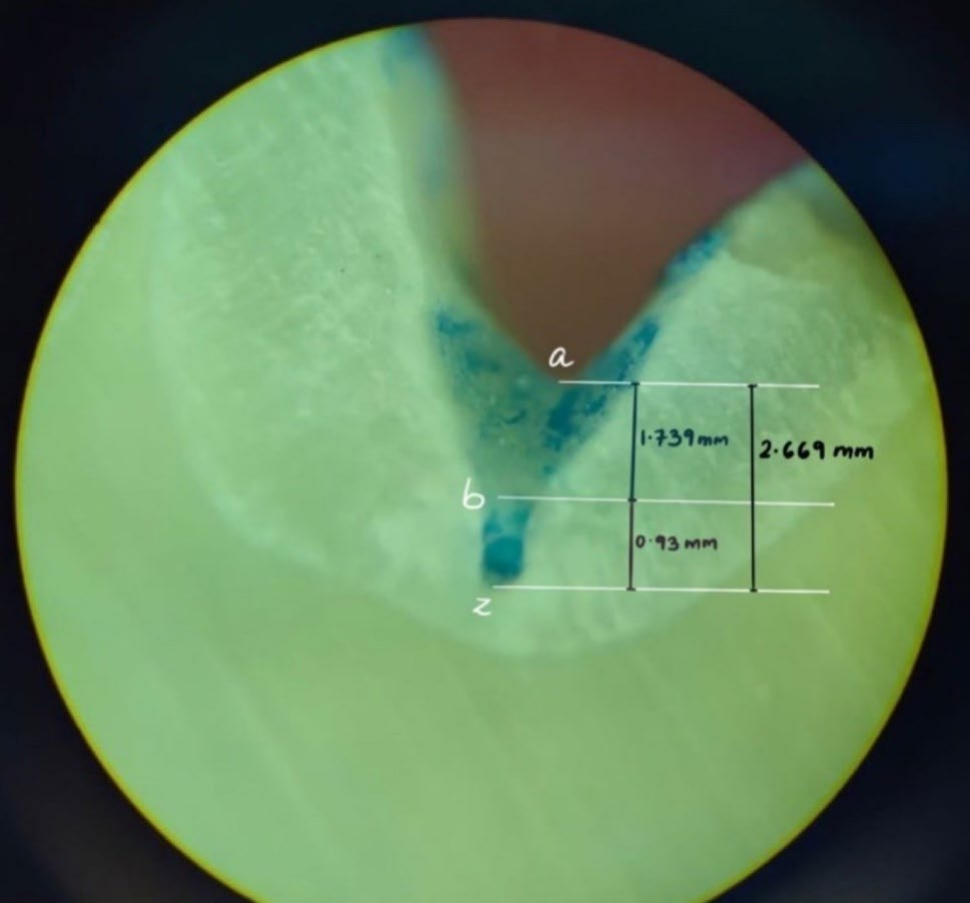
Microscopic image of a sectioned tooth with GIC sealant illustrating the measurements made to check the penetrability. The fissure morphology noted here was I type

The Image J software (National Institutes of Health, Bethesda, USA) was used to obtain all the linear measurements, which were calculated in microns (µ). For each section, three measurements were considered and their mean was taken during the analysis (Figs. 2, 3). The data were tabulated and analyzed statistically.

### Statistical analysis

SPSS version 2.0 was used for performing statistical analysis. The Chi-square test was used for the comparison of the sealing ability between the groups. The comparison of penetration was done using an Independent *t* test. The level of significance was set at 5% (*p* < 0.05).

## Results

Out of the 20 teeth used in the present study, 3 were permanent molars (2 mandibular and 1 maxillary) and the remaining 17 teeth were premolars (14 maxillary, 3 mandibular). Following the random distribution of the samples, Group I had one mandibular molar, seven maxillary premolars, and two mandibular second premolars, while Group II consisted of one mandibular molar, one maxillary molar, seven maxillary premolars, and one mandibular second premolar. The frequency distribution of various types of fissures seen among the selected teeth is shown in Table 1. Fissure types I, IK, U, and V were found to be statistically non-significantly distributed between the two groups. In the comparison of the sealant penetration, the two groups did not show a significant difference, statistically (*p* = 0.104, Table 2). Similarly, the microleakage scores between the two groups also did not show any significant difference (*p* = 1.0, Table 3).

**Table 1:** Frequency distribution of various types of fissures across two groups

	Groups		Total
	Group I	Group II	
Fissure morphology			
I			
Count	4	1	5
% Within group	40.0%	10.0%	25.0%
IK			
Count	1	0	1
% within Group	10.0%	0.0%	5.0%
U			
Count	1	6	7
% Within Group	10.0%	60.0%	35.0%
V			
Count	4	3	7
% Within group	40.0%	30.0%	35.0%
Total			
Count	10	10	20
% Within group	100.0%	100.0%	100.0%

Chi-square tests	Value	*df*	*p*-value (< 0.05 is significant)
Pearson Chi-square	6.514	3	0.089

**Table 2:** Independent *t* test to compare the sealant penetration property between the two groups

	Group I (bioactive smart material sealant) (*n* = 10) Mean ± SD	Group II (GIC sealant) (*n* = 10) Mean ± SD	*t*	*p*-value
Sealant penetration depth	0.93 ± 0.42	0.9 ± 0.72	0.099	0.922
Length of unfilled space	0.32 ± 0.6	0.02 ± 0.06	1.593	0.145
The total length of the fissure	1.38 ± 0.74	1.62 ± 1.24	− 0.536	0.6
Penetrability	0.73 ± 0.24	0.52 ± 0.3	1.712	0.104

**Table 3:** Comparison of the microleakage scores between the two groups

	Group		Total
	Group I	Group II	
Microleakage scores			
Score 0			
Count	6	6	12
% Within group	60.0%	60.0%	60.0%
Score 2			
Count	2	2	4
% Within group	20.0%	20.0%	20.0%
Score 3			
Count	2	2	4
% Within group	20.0%	20.0%	20.0%
Total			
Count	10	10	20
% Within group	100.0%	100.0%	100.0%

Chi-square tests	Value	*df*	*p*-value (< 0.05 is significant)
Pearson Chi-square	0.000	2	1.000

## Discussion

Currently, resin-based pit and fissure sealants are being popularly used worldwide. But, the resin sealants predispose to the accumulation of biofilms due to microleakage (Lin 2017; Spinell et al. 2009; Sun et al. 2009). Thus, fluoride-releasing pit and fissure sealants are more beneficial due to their anti-cariogenicity and remineralizing efficiency (Xu et al. 2006; Xu and Burgess 2003). In addition, the fluoride release from sealants can also help to enhance the hardness of underlying demineralized enamel and dentin (Sivapriya et al. 2017). Absolute isolation is also a critical factor for the retention and success of resin-based sealant materials (Sreedevi et al. 2022). But, pediatric dentistry does not vow total isolation, especially in cases where patient compliance is limited. Thus, moisture-friendly sealants and sealants requiring less number of steps during placement are more appropriate (Garg et al. 2019). Caries progression, under the sealed surface, is another concern that demands the usage of a sealant having a quintessential ability to remineralize (Netalkar et al. 2022). Keeping in mind the aforementioned factors, in the present study, we explored the possibility of  using ACTIVA BioACTIVE-Base/Liner, which is bioactive, fluoride-releasing, moisture friendly, and has also got remineralizing potential as a pit and fissure sealant.

ACTIVA BioACTIVE Base/Liner is indicated to be used as an alternative to all types of glass ionomer cements, and flowable composites and can be used without etchants or bonding agents, thereby offering a quick chairside procedure (Vouzara et al. 2020). It is a “light-cured resin-modified calcium silicate”, manufactured as an uncompromised blend of the uniqueness of composites (strength, esthetics, and physical properties) and glass ionomer cement (fluoride release) (Kunert and Lukomska-Szymanska 2020). Chemically, the material consists of Bioactive glass as a filler in a diurethane and methacrylate base with a modified polyacrylic acid and polybutadiene-modified diurethane dimethacrylate (rubberized resin) (van Dijken et al. 2019). During critical pH, this material is capable of releasing and recharging ions such as calcium, phosphate, and fluoride in greater quantum than the glass ionomers. Thus, it can efficiently stimulate apatite crystal formation, thereby helping remineralization. Additionally, the strong and resilient resin matrix of this bioactive cement does not chip or crumble. ACTIVA BioACTIVE Base/Liner is biocompatible and is free of Bisphenol A, Bis-GMA, and BPA derivatives (Karabulut et al. 2020).

One of the ideal requisites of a material to be used as pit and fissure sealant is obtaining a good seal (Garg et al. 2019). Therefore, we studied microleakage as one of the properties when ACTIVA BioACTIVE Base/Liner is used as a sealant. We used the methylene blue dye penetration test, which is not only an inexpensive and easily accomplished test but is also known to be a reliable method of testing microleakage at the sealant margins (Agrawal and Shigli 2012; Kramer et al. 2008). While the method is associated with the concerns of acute methylene blue toxicity upon oral intake (Amend et al. 2021), it should be noted that the present study was carried out in vitro.

We found no difference in the microleakage of the tested material in comparison to glass ionomer sealant. Jain et al. (2022) also have found no difference in the microleakage scores between ACTIVA BioACTIVE and glass ionomer cement. Similarly, Kaushik and Yadav (2017) also reported no statistical difference in the microleakage of ACTIVA BioACTIVE compared to that of nanohybrid composite. On the contrary, a study by Tohidkhah et al. (2022) showed that ACTIVA BioACTIVE exhibited higher microleakage than incrementally filled composites and resin-modified glass ionomer cement. A common observation was made in the studies by Kaushik and Yadav (2017) and Tohidkhah et al. (2022) that etching and applying a bonding agent while restoring the tooth using Activa Bioactive significantly reduces microleakage. Interestingly, the study by Jain et al. (2022) also etched the tooth and applied a bonding agent while placing the restoration. However, all these studies have used ACTIVA BioACTIVE restorative, while the present study has used ACTIVA BioACTIVE Base/Liner. In the present study, etching was not done before sealing the tooth using ACTIVA BioACTIVE Base/Liner. We followed the manufacturer’s instructions for placement.

For a material to be used as pit and fissure sealant, most importantly, it should possess good flowability to enable its penetration into the deep pits and fissures completely thereby establishing a good mechanical barrier (Cvikl et al. 2018). Thus, another parameter we chose to evaluate in the present study is penetration. ACTIVA BioACTIVE Base/Liner has got a film thickness of about 11 µm when mixed as per the manufacturer’s recommendations. Our study showed statistically no difference in the penetration depth of ACTIVA BioACTIVE Base/Liner in comparison to glass ionomer sealant. The fissure pattern is known to influence the penetration depth of the sealant. Muntean et al. (2019) reported the greatest penetration of the sealant in U-type fissures and the least in I-type fissures. Thus, we evaluated the fissure pattern distribution in each group and it was found that there was no difference in the distribution of different fissure patterns between the groups.

Activa bioactive smart material is a recent addition to the dental materials available in the market and to the best of our knowledge, research exploring ACTIVA BioACTIVE Base/Liner is limited. The findings of the present study accept the set null hypothesis, however, within the following limitations. This is a pilot study conducted in vitro evaluating only two important properties of a pit and fissure sealant. There are many other critical factors that a sealant should possess apart from penetration and microleakage, which were not evaluated in our study. Also, this study compared the tested properties of the ACTIVA BioACTIVE Base/Liner smart material to glass ionomer sealant and not the resin-based sealant. In addition, in the present study, we did not standardize the sample to any one type of tooth. We used both molars and premolars. Thus, future studies have to be conducted exploring ACTIVA BioACTIVE Base/Liner as a pit and fissure sealant.

## Conclusion

Within the limitations of the study, the Bioactive Material as a pit and fissure sealant, performed on par with glass ionomer sealant in terms of tested properties like sealing ability and penetration.

## Data Availability

Raw data (master table) is available upon request.
